# Special Issue “Repetitive DNA”

**DOI:** 10.3390/ijms27104402

**Published:** 2026-05-15

**Authors:** Eva Šatović-Vukšić, Manuel A. Garrido-Ramos, Miroslav Plohl

**Affiliations:** 1 Division of Molecular Biology, Ruđer Bošković Institute, Bijenička 54, 10000 Zagreb, Croatia; miroslav.plohl@irb.hr; 2 Departamento de Genética, Facultad de Ciencias, Universidad de Granada, 18071 Granada, Spain; mgarrido@ugr.es

The Special Issue entitled “Repetitive DNA” highlights the growing recognition that repetitive sequences are fundamental components of genome biology. Repetitive DNAs constitute a substantial fraction of eukaryotic genomes, yet for a long time they remained underestimated, often labeled as non-functional “junk.” This view has fundamentally changed over the past decades (reviewed in [1,2,3]). Today, satellite DNAs (satDNAs) and transposable elements (TEs) are recognized as essential contributors to genome architecture, chromatin organization, gene regulation, and evolutionary processes (reviewed in [4,5,6,7,8]). The shift in the paradigm has been driven largely by methodological advances, particularly in long-read sequencing and high-throughput bioinformatic pipelines, which have enabled insights to repeat-rich genomic regions that were previously difficult to resolve (reviewed in [9,10,11]). As a result, the study of the satellitome and the repeatome has become central to modern genomics. At the same time, complex dynamics in which repetitive elements are both drivers and products of genome evolution are becoming more evident (reviewed in [4,5,12,13,14]). For example, repetitive DNA plays essential roles in defining chromosomal architecture, particularly in centromeric and pericentromeric regions where satDNA contributes to kinetochore formation and chromosome segregation. Functionally, repetitive DNA is increasingly recognized as an active participant in genome regulation. SatDNA transcription has been linked to chromatin organization, centromere function, and stress responses (reviewed in [12,13]). In parallel, TEs have shown to be major contributors to genomic regulatory processes, providing enhancers, promoters, and alternative splice sites, as well as participate in genome rearrangements, gene silencing or even tail loss in Hominoidea [4,15]. Moreover, these sequences are not confined to heterochromatin, and their presence in euchromatic regions has been increasingly documented and discussed (reviewed in [3]). The role of repetitive DNA is also very important in genome evolution. Studies based on variety of eukaryotic organisms have demonstrated that repeat expansion, contraction, and diversification are key drivers of genome size variation, chromosomal rearrangements, species divergence, and chromosome evolution (chapters within [2,16]).

The contributions gathered in this Special Issue reflect the wideness of the research field of repetitive DNA biology, spanning functional analyses and evolutionary studies, all supported by experimental, bioinformatic, and contemporary sequencing methodologies. To provide a coherent overview, the articles in this Special Issue can be organized into interconnected thematic groups, beginning with broader conceptual framework, followed by functional insights, and continuing into genome organization and evolution studies.

The conceptual framework is set with a review by Garrido-Ramos et al. (contribution 1). This contribution synthesizes recent advances to demonstrate that satDNAs are dynamic, diverse, and functionally relevant components of genomes. It covers the diversity of repeat families, and the complexity of their intraspecies and interspecies distribution patterns. The review also integrates some basic features of different forms of sequences repeated in tandem, along with rapidly growing data evidencing extensive dispersal of satDNA sequences in euchromatin, their putative roles, and evolutionary significance. Central to this discussion is the concept of the satellitome, which encompasses the full complement of satDNAs within a genome. Advances in sequencing technologies and bioinformatic pipelines have made it possible to systematically characterize satellitomes across species, revealing their diversity, rapid evolution, and species-specific patterns. Importantly, the review points out various issues brought on by the use of new methodological approaches and discusses potential threats—accenting those which could lead to misleading interpretations of repeat composition, localization, abundance and evolutionary mechanisms. By integrating structural, functional, and evolutionary perspectives, this review puts into context the following research articles in the Special Issue.

The first set of contributions addresses the functional roles of repetitive DNA, demonstrating its active involvement in gene regulation and genome stability. Ljubić et al. (contribution 2) show that alpha satDNA, traditionally associated with centromeres, can exert regulatory effects at the transcriptional level. Their work demonstrates that overexpression of alpha satellite RNA leads to the downregulation of genes containing homologous intronic repeats, likely mediated through RNA–DNA hybrid formation. This finding positions satDNA transcripts as active participants in gene regulatory networks, playing a role in modulating the expression of alpha satDNA-associated genes. In a complementary study, Merkulov et al. (contribution 3) examine the consequences of TE mobilization in *Arabidopsis thaliana*. They demonstrate that DNA methylation and alternative splicing act as rapid buffering mechanisms following a burst of retrotransposition of ONSEN elements. Many insertions are “intronized” and incorporated into transcripts without disrupting gene function. The ONSEN insertions provided alternative transcription start or termination sites, generating novel transcript isoforms, allowing repurposing of novel repetitive DNA sequences. These two studies reinforce the idea that repetitive DNA is functionally integrated into genomic processes, from the aspects of regulatory processes and genomic resilience.

The next group of research papers focuses on the structural organization of repetitive DNA within plant genomes, combining sequencing-based analyses with cytogenetic techniques. Muravenko et al. (contribution 4) investigate the repeatome of *Polemonium caeruleum*, identifying a predominance of retrotransposons alongside multiple satDNA families. This approach allowed them to map intra-species chromosomal variability and rearrangements linked to the speciation of *P. caeruleum*. Furthermore, these new markers can help clarify the taxonomy and phylogeny of *Polemonium* and provide valuable resources for plant breeding programs. Similarly, Amosova et al. (contribution 5) examine four species of the genus *Amaranthus*, reporting variation in the content and the amount of repetitive sequences. Their comparative analysis, supported by cytogenetic mapping, enables the reconstruction of karyotypes and contributes to resolving phylogenetic relationships within the genus. Kalnyuk et al. (contribution 6) attend repeatome diversity in the genus *Salvia*, linking repetitive DNA dynamics to polyploidy, chromosomal rearrangements, and taxonomic complexity. This work bridges structural and evolutionary perspectives, highlighting repetitive DNA as a major driver of genome diversification. These three contributions illustrate how repeatome analysis, combined with cytogenomics, enables characterization of chromosomal architecture and comparative analyses across species, enabling development of tools that are of use in taxonomy, phylogeny and beyond.

Closely related to genome organization is the role of repetitive DNA in chromosome evolution, particularly in rapidly evolving systems such as sex chromosomes. Barcellos de Oliveira et al. (contribution 7) employ cytogenetics and genomics tools to examine the composition of sex chromosomes through characterization of repeatome and sex-linked markers systems in the fish *Hoplias malabaricus*. They show that while overall repeated content remains similar, specific satDNA variants accumulate in non-recombining regions of the Y chromosome. This localized expansion contributes to differentiation between sex chromosomes. Setti et al. (contribution 8) investigate several snake genomes and demonstrate that W chromosomes are enriched in satDNAs and microsatellite repeats. Their findings indicate that repeat accumulation does not always correlate with heterochromatin, pointing to multiple evolutionary trajectories in sex chromosome differentiation. Together, these studies underscore the central role of repetitive DNA in shaping chromosome evolution, and its involvement in the emergence and differentiation of specialized chromosome systems.

The contributions to this Special Issue collectively demonstrate that repetitive DNA is central to genome function and evolution. From regulating gene expression and buffering genomic instability, to shaping chromosome architecture and driving evolutionary processes, repetitive sequences are actively involved in multiple levels of genome organization. The works presented here represent a cross-section of the current research directions in repetitive DNA research field. As sequencing technologies continue to advance, the study of repetitive DNA is entering a new era of resolution and insight. In this ongoing story, integrative and interdisciplinary approaches will be essential for fully understanding the complexity and reach of repetitive DNAs.
